# Autonomous control for miniaturized mobile robots in unknown pipe networks

**DOI:** 10.3389/frobt.2022.997415

**Published:** 2022-11-16

**Authors:** T. L. Nguyen, A. Blight, A. Pickering, G. Jackson-Mills, A. R. Barber, J. H. Boyle, R. Richardson, M. Dogar, N. Cohen

**Affiliations:** ^1^ School of Computing, University of Leeds, Leeds, United Kingdom; ^2^ School of Mechanical Engineering, University of Leeds, Leeds, United Kingdom; ^3^ Faculty of Industrial Design Engineering, Delft University of Technology, Delft, Netherlands

**Keywords:** in-pipe robot, autonomous control, infrastructure robot, exploration, water & sewer pipes, navigation, exhaustive search, miniature robot

## Abstract

Despite recent advances in robotic technology, sewer pipe inspection is still limited to conventional approaches that use cable-tethered robots. Such commercially available tethered robots lack autonomy, and their operation must be manually controlled *via* their tethered cables. Consequently, they can only travel to a certain distance in pipe, cannot access small-diameter pipes, and their deployment incurs high costs for highly skilled operators. In this paper, we introduce a miniaturised mobile robot for pipe inspection. We present an autonomous control strategy for this robot that is effective, stable, and requires only low-computational resources. The robots used here can access pipes as small as 75 mm in diameter. Due to their small size, low carrying capacity, and limited battery supply, our robots can only carry simple sensors, a small processor, and miniature wheel-legs for locomotion. Yet, our control method is able to compensate for these limitations. We demonstrate fully autonomous robot mobility in a sewer pipe network, without any visual aid or power-hungry image processing. The control algorithm allows the robot to correctly recognise each local network configuration, and to make appropriate decisions accordingly. The control strategy was tested using the physical micro robot in a laboratory pipe network. In both simulation and experiment, the robot autonomously and exhaustively explored an unknown pipe network without missing any pipe section while avoiding obstacles. This is a significant advance towards fully autonomous inspection robot systems for sewer pipe networks.

## 1 Introduction

Sewer systems are a crucial element of urban infrastructure. They not only transport sewage to processing centers but also reduce flood risk for the cities. However, sewer systems are exposed to harsh conditions such as mixture chemical reaction, excessive traffic, geological change, earthquakes. Pipes may suffer from corrosion, damage, deformation, leakage, siltation, obstruction, and other defects due to long-term deterioration. These defects will reduce the efficiency of the sewer system and in extreme cases sewage may leak out, polluting soil and ground water. Leaked sewage may also wash away soil, eroding the foundations of buildings or street pavements, possibly causing surface collapse or weakening foundations. Thus, sewer inspection is required extensively and regularly to provide early detection of defects and hazards. From there, hazard prevention measures, maintenance and upgrades can be planned and provided.

Today, sewer inspection still heavily relies on a manual field survey (Chuang and Sung, 2020), in which workers have to stay underground to inspect the sewer system. This method involves potential hazard and health risks to the human workers due to under-ventilated and harmful gas environment. It is also difficult and tedious, taking a long time and requiring highly trained inspectors (Chuang and Sung, 2020). In addition, the majority of drain/sewer pipes are too small for human inspectors to enter (most sewer pipes are smaller than 400 mm in diameter (Drainagesuperstore, 2022)). To address these issues, in the last few decades, specific robots have been developed to replace human entry for inspection of buried pipes (Scholl et al., 2000; Nassiraei et al., 2007; Kim et al., 2010; Wang et al., 2010; Lee et al., 2011; Lee et al., 2012; Qiao et al., 2013; Debenest et al., 2014; Yum et al., 2014; Song et al., 2016; Knedlová et al., 2017; Hayat et al., 2018; Li et al., 2018; Mills et al., 2018; Kakogawa and Ma, 2019; Zhao et al., 2020; Brown et al., 2021; Virgala et al., 2021; Jang et al., 2022; Minicam, 2022).

However, most of these robots are under development and commercial inspection robots are still limited to conventional approaches which use tethered crawlers (Minicam, 2022) with an onboard video camera system. In this state-of-the-art technology, the human operators insert the robotic crawler along with its tethered cables into the pipe *via* the manhole. After that, the robot is manually controlled *via* the cables to crawl into the pipe and the inspection camera is remotely operated to record a video inside the sewer system. Video footage will then be analyzed offline to detect defects. Although such a tethered crawler can provide stable remote control, continuous power for strong motors, long operational time and real-time video streaming to the central station, the robot has to pull the long cables behind it. These cables are heavy and often stiff causing poor mobility to pass any kind of pipe-bends such as curves and junctions. As a result, these robots are generally only capable of moving along straight pipe sections. Thus, after the inspection of a pipe section between two manholes, the robot and cables must be pulled back and re-deployed at the next manhole. This method for inspection of the sewer pipes makes the inspection process very slow and costly (Nassiraei et al., 2007).

Effective autonomous pipe inspection robots would reduce the cost and time expense compared to current methods, hence improving the inspection efficiency. The robot must be an un-tethered mobile robot, equipped with inspection sensors such as video, acoustic, ultra-sound and navigation systems (odometry, simultaneous localization and mapping) for completely autonomous operations in buried pipes. To address this challenge, the Pipebots project (Pipebots, 2022) aims to develop robotic platforms for autonomous inspection of underground pipe networks. For effective, scalable and versatile operation underground, we envision a collaboration between multiple robot platforms in which medium size robots inspect medium (250–400 mm) and large (450–900 mm) sewer pipes and micro-robots reach into the smaller sewer tributaries (75–200 mm).

As the most common size of sewer pipes is 100 mm in diameter (NewFlow-Inc, 2022), Pipebots (Pipebots-Theme3, 2022) had designed a micro-robot (hereafter dubbed Joey, because it is intended to be carried and deployed by a larger robot) for inspection of these smaller sewer pipes. Joey robots are equipped with micro motors, compact and lightweight shells, a battery and a self-contained electronics payload on custom PCBs. They are able to operate in pipes as small as 100 mm diameter. Although Joeys can carry an extra extension circuit with a camera and three ultra-bright LEDs for visual inspection and visual aid in localization, these components and high-computational image processing rapidly drain the robot battery. Consequently, like most other small robots, Joeys suffer from a short battery life. Thus, any autonomous control algorithms of these robots must address both locomotion and navigation challenges intelligently and efficiently to complete tasks in a short time and minimizing power consumption.

In this paper, we introduce the Joey robot as well as an effective control method for autonomous pipe exploration. To evaluate the performance of the system, the Joey robot is deployed autonomously in a small diameter (150 mm) sewer pipe network. The 3D-printed robot runs the control algorithm on its on-board processor and relies on low-energy sensors namely: two low-resolution encoders (that are connected to the motors driving the left and right sets of wheel-legs); three laser-based range sensors installed at the robot front; and an IMU (inertia measurement unit) on the back of the robot. The processing unit is a low-power microcontroller (STMicroelectronics) with maximum clock speed of 64 MHz. The pipe network is a laboratory mockup of a typical underground plastic sewer pipe network built at ICAIR (the Integrated Civil and Infrastructure Research Centre, University of Sheffield). In this paper, the goals include an autonomous exhaustive exploration of a given pipe network, and a safe return to the starting point without flipping, getting stuck, or depleting the battery. In our experiments, a Joey robot is able to complete the exhaustive exploration and return safely within 4 min in a 5-m-long pipe network with complex geometry.

The remainder of this paper is organised as follows: section 2 introduces the robot and its design for operation in sewer pipes; section 3 presents the navigation strategy, control algorithm, and its implementation; section 4 describes the experimental setup and results, followed by section 5 in which conclusions and future work are discussed.

## 2 Miniaturized robot in sewer pipes

### 2.1 Robot design and locomotion

**FIGURE 1:**
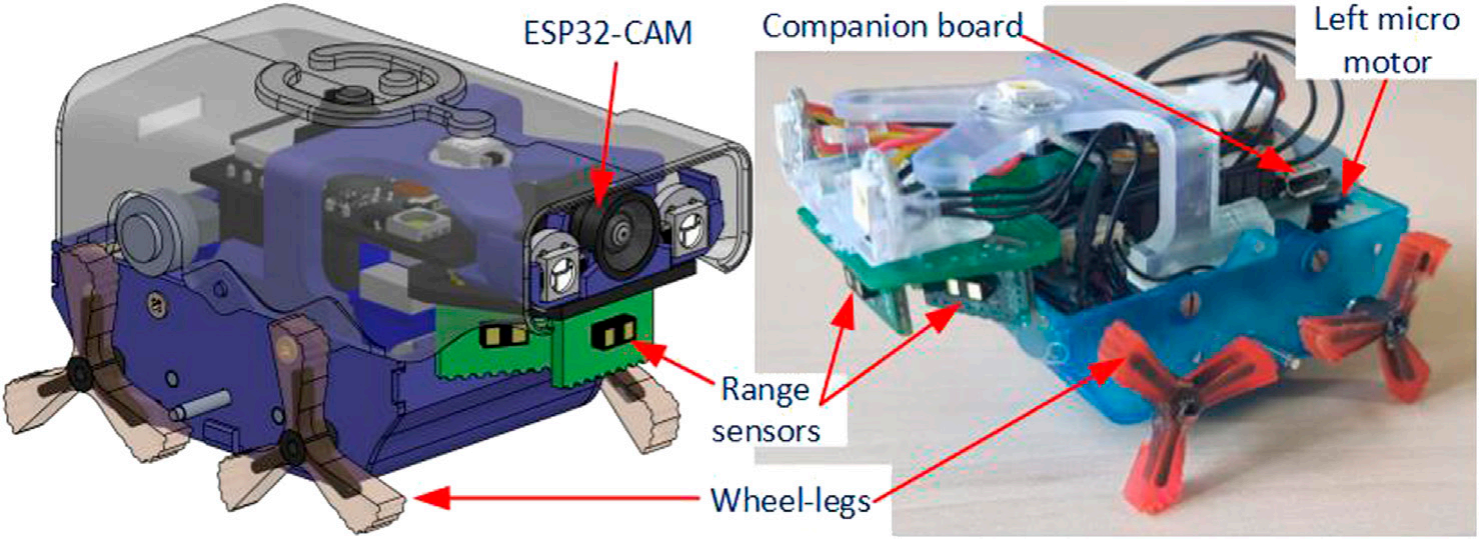
Robot CAD model and current physical model used in this paper. The main difference between the two models is that the physical robot does not need/equip an ESP32-CAM (development board for streaming camera video *via* WiFi) and has a simplified upper shell (for convenient downloading of the program for experiments).

The Joey is designed to operate in small sewer or drainage pipes (as small as 100 mm diameter). It features wheel-legs for locomotion, to enable the robot to traverse obstacles and slippery terrains. The wheel-legs allow the robot to “roll” like other wheel robots and “walk” over small rocks, pebbles, low steps in the pipes, manholes, and uneven junctions. Figure 1 shows the CAD model (Pipebots-Theme3, 2022) and physical robot used in this paper. The robot total width is 54 mm and total length is 74 mm. Its total weight with full ESP32-CAM extension and battery is about 70 g.

The Joey is equipped with two micro-motors, each of which actuates all the wheel-legs on either the left or right side of the robot *via* a set of spur gears. As a result, the robot uses a skid steering method (Shuang et al., 2007) to change its direction. To reduce the robot size, the small (6 mm diameter and 21 mm length) DC gearmotors (Precision MicroDrive: 206-10C) are used and a motor driver chip 206TDRV8833 is integrated on the main circuit board (called Companion board). As it is not possible to mount an encoder on the current micro-motor, a magnetic encoder (Pololu 4760) is mounted on the driveshaft of one wheel-leg on each side and used for odometry and motor control. The robot can move both forward and backward at a maximum speed of 70 mm/s.

**FIGURE 2:**
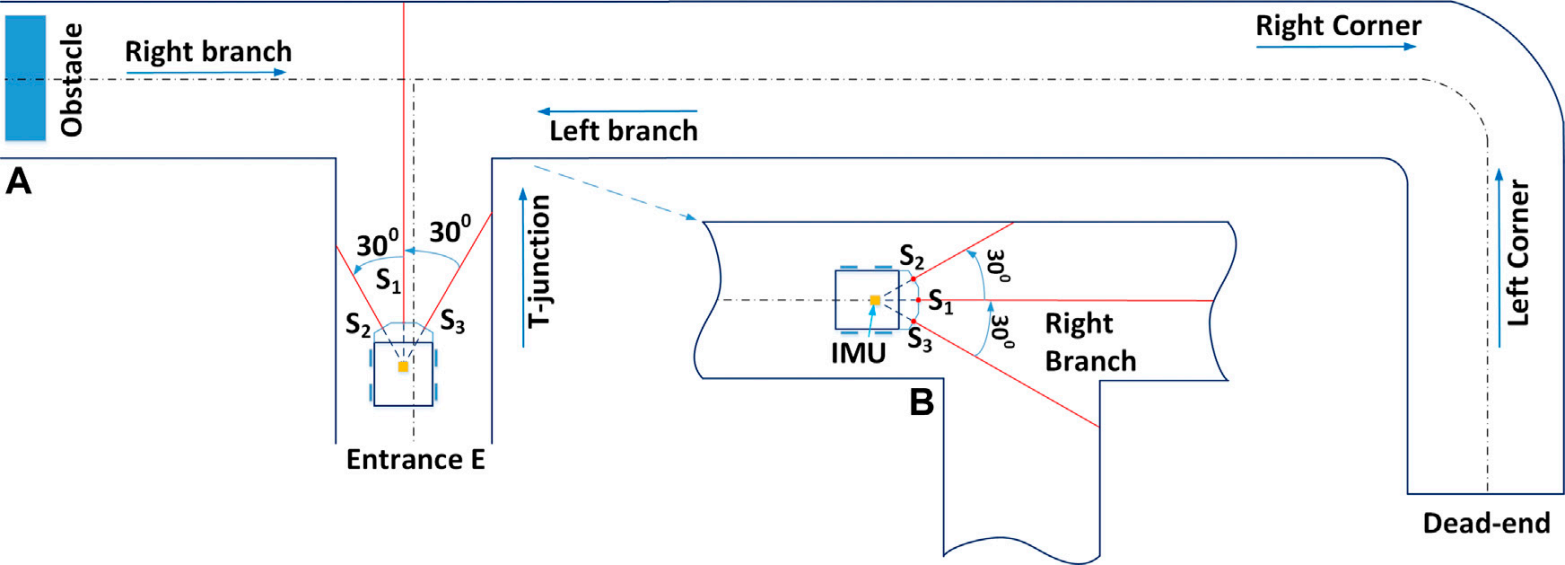
Diagrams of Joey robot operating inside a schematic of our experimental pipe network. Panel **(A)** depicts the Joey robot at starting point (Entrance E) of a typical imitation in laboratory setting of a real sewer pipe network at the Integrated Civil and Infrastructure Research Centre, University of Sheffield (ICAIR). The imitated pipe network includes features of the real network such as T-junction, corners, branches, dead-end and obstacle. Panel **(B)** illustrates the robot coming from the left side of the network in Panel **(A)**. Panel **(B)** also illustrates the left/right ranging direction which form angles of 30° with robot front-back axis.

In addition to the encoders, Joey robots are equipped with three time-of-flight range sensors (VL53L1X from STMicroelectronics - Pololu 3415), an IMU (ICM-20948 from InvenSense) and a MEMS microphone (IMP34DT05 from STMicroelectronics). The range sensors are placed in front, front left, and front right of the robot (as shown in Figure 2A at locations S_1_, S_2_, S_3_ respectively). These range sensors measure distance to nearest points at straight front direction, and at 30° to the left and right of straight front direction. Figure 2 shows the ranging directions of these sensors relative to the robot front-back axis. In addition, the robot Companion board has connections to an extension board ESP32-CAM (AI Thinker) which is mounted on top of the Companion board, providing camera streaming *via* 2.4 GHz Wi-Fi communication. To support camera vision, the robot is also equipped with three ultra-bright LED NeoPixels (WS2812 from WorldSemi) light sources.

In term of central processing units, the Companion board was designed with an STM32WB5MMG processor (from STMicroelectronics). This is an ultra-low-power microcontroller supporting Bluetooth^®^ Low Energy 5.0, Zigbee^®^ 3.0, and OpenThread communications at certified 2.4 GHz. These communication supports will be used for collaborative and swarm control in future. However, it is a low-power microcontroller with a main core maximum speed of up to 48 MHz and a dedicated float processing unit of 64 MHz, with limited RAM of 256 kB. Therefore, the main processor is not designed for heavy computational work such as machine learning or image processing algorithms.

In summary, the miniature Joey robot is entirely autonomous, and operates without any tethered cables. When tested in a 100 mm pipe section/network, the Joey successfully walks while monitoring its accelerations and orientations with the integrated IMU, monitor the distances from the robot’s front to the closest straight front, front-left and front-right pipe wall/object with range sensors, monitor the left/right wheel-leg rotation with encoders, and record videos of the pipe conditions with on board camera and SD card. All recorded video and data can then be post-processed on a computer for pipe condition monitoring, asset mapping, maintenance, and upgrade.

### 2.2 Sewer pipe inspection tasks

Joey robots are designed to work in hard-to-reach small sewer pipes. They have three main tasks:

**Task 1:** To explore small pipe networks while performing a basic inspection.
**Task 2**: To collect/record and process inspection data (leakage or blockage information: location, dimension, material, changes, images/videos; pipe conditions: rust sign, color, images/videos; assets map).
**Task 3**: To collaborate and communicate with other robots.

This paper focuses on the first task which is to autonomously explore an unknown pipe network. We assume the pipes are empty before inspection, we use clean, dry pipes. The robot must operate without any cable, remote control, or human intervention, and exhaustively traverse the entire pipe network without missing any section. The robot must autonomously decide (in real time) how to move in order to perform this navigation task without access to a map, GPS data, video or acoustic inputs. Figure 2A illustrates a Joey robot autonomously navigating in a sewer pipe network. The robot enters the pipe network at entrance/exit E, exhaustively explores the whole network and returns to exit/entrance E. Depending on the robot position and orientation, it would face different pipe junctions from different perspectives. The sensors allow the robot to distinguish between these configurations. For instance, when the robot first enters at entrance E, it “sees” the junction ahead as a T-junction (Figure 2A) but when the robot walks from the obstacle towards the same junction, it “sees” it as a right-branch (Figure 2B). The distinction is important for the exploration protocol (see section 3.2 below).

In addition to the above-described sensors, the Joey has a camera and light source. To reduce power-consumption and thus increase operation time, in an accompanying paper we propose only to switch on the camera and light source only when those are essential for inspection and mapping (Li et al., 2022). When operational, the camera takes images and videos, and saves them to an SD card. In the current prototype, the camera, extension board, any image processing algorithm, and streaming are not used during live operations. These are not turned on or used for the control of the Joey in this paper.

### 2.3 Challenges of robot control in small sewer pipes

As explained in previous sections, the first challenge in autonomous control of the Joeys in sewer pipes is implementing a robust protocol for the execution of task 1 with only simple, low-power onboard sensors (three front range sensors, one integrated IMU, and two encoders attached to the drive system).

The second challenge is to overcome physical flaws of Joey robots which are the design sacrifices to achieve miniaturized robot able to operate in small spaces. Six physical limitations and their consequences for robot mobility and stability are discussed here: First, as the pipe is not flat, the robot must constantly stabilize its motion using the encoders. But because the small and low-cost encoders cannot be mounted on the micro motor shafts, they are attached to the output side (i.e., the wheel-leg shaft). Thus, a key constraint is the low sampling rate of the encoders: there are only a total of 12 pulses with quadrature reading mode for each revolution of the wheel-legs. Second, the initial angular position of the six wheel-legs is designed and assembled so that only three wheel-legs are in contact with the flat floor while the other three wheel-legs are in opposite phase (the alternating triplets consist of the front and back wheel-legs on one side and the middle wheel-leg on the other side). This tripod gait would maintain the balance for the robot base. However, in a physical robot, the left and right wheel-legs are often out of sync as a consequence of the 3D printed gear-set backlash. The limited tolerance can destabilize the robot body during locomotion, which in turn may result in deviations from the intended direction along the robot trajectory even when robot is walking on flat surface. The challenge to autonomously maintain the robot balance is exacerbated when the robot is carrying out a maneuver or moving in a pipe, or other inclined surfaces. Third, Joeys use differential drive for steering. As the wheel-legs on each side are geared together, some slipping is inevitable during turning maneuvers. Slipping interferes with odometry measurements and hence with closed-loop speed/position control. Fourth, as there are multiple spur gears connecting the micro-motor and wheel-legs and the current Joey version uses 3D printed plastic gears, there is significant backlash causing high overshoots for position control. Fifth, related to this gear backlash, when the wheel-legs are out of sync, robot locomotion makes its body wobble vertically, causing unstable readings on the range sensors and IMU. Finally, as the robot center of mass is quite high, the robot faces significant flipping risk. The prototype Joeys used in this paper are not able to recover once flipped. These challenges must be overcome even when the pipes are perfectly clean and horizontal. In this paper, we first test autonomous deployment of the robot in such ideal conditions and then confront the robot with additional challenges, including inclined pipes and liquid or granular substrates on the pipe interior (Section 4).

## 3 Autonomous control algorithm

### 3.1 Autonomous navigation strategy

**FIGURE 3:**
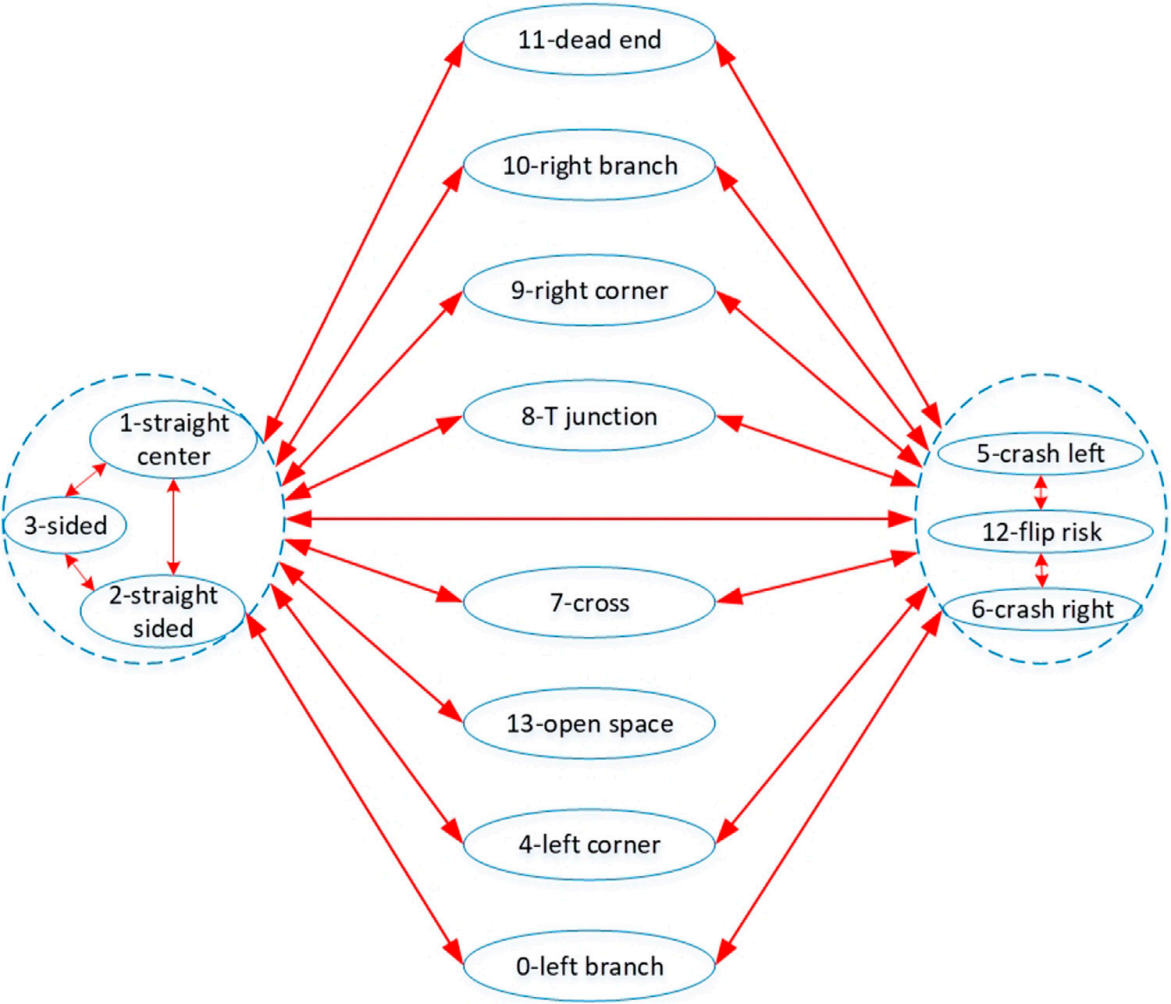
Possible robot states in sewer pipe networks. Each arrow represents a possible state transfer. Junction-like states (i.e., branches, cross, corners, dead end) do not transfer directly to each other because of their distance from each other in real sewer pipe networks. They often interchange to straight pipe states, or sided states during autonomous navigation. These three states are interchangeable but have similar conditions thus are grouped together. Another group includes the risk states (i.e., crash risks, and flip risk). The risk states often occur during maneuvering at junctions.

To overcome the challenges described in section 2.3 and to ensure the execution of Task 1 described in section 2.2, we propose a computationally cheap, autonomous control strategy based on a finite state machine. By considering all possible events that a Joey may encounter within a sewer pipe network, we defined 13 robot states. We assume that the robot can confirm its current state using its sensor readings. Given its current state, the robot will call the corresponding high-level decision-making protocol to operate autonomously. The robot states are described in Figure 3. These states include:

**Straight center** and **straight sided**: The robot is in a straight pipe section, but the former is when robot front-back centerline is “close” to the pipe centerline while the latter is when robot front-back centerline is far from the pipe centerline. The definition of “close” is:

$$\frac{R_{left}-R_{right}}{R_{left}+R_{right}}\leq k_{c}$$ (1)

where $R_{left}$ and $R_{right}$ are of the left and right range sensor readings, and $k_{c}$ is a defined limit.

**Sided**: This state is similar to **straight sided** except its front range value is under a defined limit *F*_*s*_ (the robot front is close to the pipe wall or obstacles). This state is often encountered during fine maneuvers and used in the control algorithm to keep the robot front-back centerline parallel to the pipe centerline or to steer the robot away from obstacles or from the pipe wall.

**Left corner** and **right corner**: The robot is at a left or right corner from its front view direction.

**Crash left** and **crash right**: The robot is about to crash to the pipe wall, dead-end wall, or obstacle. “Left” or “right” denotes the side from which the robot has rotated towards the crash object/wall. These two states provide limits for the robot as it steers and allow it to find stable states without crashing or flipping.

**Flip-risk**: The robot has either detected a risk of flipping over if it moves forwards or backwards, or it has detected an over-inclined orientation risking flip sideways. This state requires fine steering out of this high-risk state.

**Dead-end**: The robot is at a dead-end in the pipe network. The robot should turn around to exhaustively explore the whole pipe network.

**T junction, left branch, right branch**: These three states refer to different perspectives of the same junction in the pipe network that depend on the robot’s direction of approach (e.g., Figures 2A,B). Based on the state, the robot decides which direction to maneuver towards.

**Open space** or **manhole**: Robot detects long ranges on all three range sensors.

**Cross**: The robot is approaching a cross junction. It should detect long output distances on both left and right range sensors, but these range values are smaller than a maximum side pipe limit.

As stated in section 1, the control inputs do not involve any simultaneous localization and mapping (SLAM) or other video or acoustic input. Instead, range and odometry sensors must suffice to confirm the robot state, and knowledge of the current state (without knowledge of robot location in the pipe network) must suffice to follow the pre-defined high-level directives. The strategy is to exhaustively cover the given pipe network and return safely to the starting point. As the robot is moving through the pipe network, the protocol allows for periodic brief activation of the camera and LED light sources to inspect the pipe conditions along the pipe sections and at junctions. This strategy ensures the execution of task 1 (section 2.2). In this paper, the ESP32-CAM is not available, and we only focus on the autonomous exhaustive exploration of task 1. To do that, there are two basic pre-defined directives for robot to operate:

- Rule 1: At junctions, turn into the furthest right direction.
- Rule 2: At dead-end, turn around to return to the previous junction.

In addition, robot states are divided into three groups which are the turning right group, turning left group, and going straight group. Also, there are two closed-loop control modes governing robot motor control:

- In straight pipes or at a left branch, the robot uses closed-loop speed control for smooth continuous rotation of wheel-legs at high speed to increase the explored area and reduce exploring time.
- Other robot states require fine robot maneuver; thus, the robot uses closed-loop position control for the left and right wheel-leg control.

There are also lower-level rules that govern the robot autonomy, maneuver, and speed. These rules will be discussed in section 3.3.

### 3.2 State estimation

The state of the robot in the sewer pipe network is estimated by a fusion of historical data of distance values from three range sensors, orientation values from the IMU, wheel-leg rotation, and previous robot states. An initial state is estimated based on range values to the nearest objects/pipe walls. If a new state (i.e., different to last state) is detected, the robot stops to re-assess its sensor values and double check the state. This process is carried out at the next cycle of state estimation and high-level control. This cycle is called the high-level control cycle and the cycle frequency can be increased or decreased depending on the robot’s state to adapt to fine maneuvers at complex junctions or fast forwards in straight pipes. If a robot state is confirmed (i.e., *state_confirm* value is larger than 1), the robot carries out the corresponding maneuver. Table 1 presents the high-level decision and action corresponding to the estimated stateFigures 4B,C. The magnitude and direction of the maneuver are defined in section 3.3 and calculated by the high-level controller based on the confirmed state. Thus, state estimation is crucial for robot operation.

**TABLE 1:** High level control actions following state estimation.

State number	State	Action
3	Sided	robot turns away from obstacles or pipe walls using fine step steering and closed-loop position control
4	Left corner	robot turns left 90° while maintaining its center near the center of the junction
5	Crash left	robot turns 30° backwards to previous turning direction, then re-evaluates its state and makes decision according to the new state
6	Crash right	
7	Cross	robot turns 90° to the right while maintaining its center near the center of the junction
8	T-junction	
9	Right corner	
10	Right branch	robot moves forwards a certain distance, turns right an angle of 45°, then, reassesses its state
11	Dead-end	robot turns around by 180° and adjusts its position to the center of pipe while maintaining its pitch and roll angles low to avoid flip risk
12	Flip risk	robot steps slowly backwards or forwards depending on its pitch and roll value to escape the flip risk
13	Open space	robot approaches slowly, reassesses its state (and optionally, monitors conditions)
	Manhole	

**FIGURE 4:**
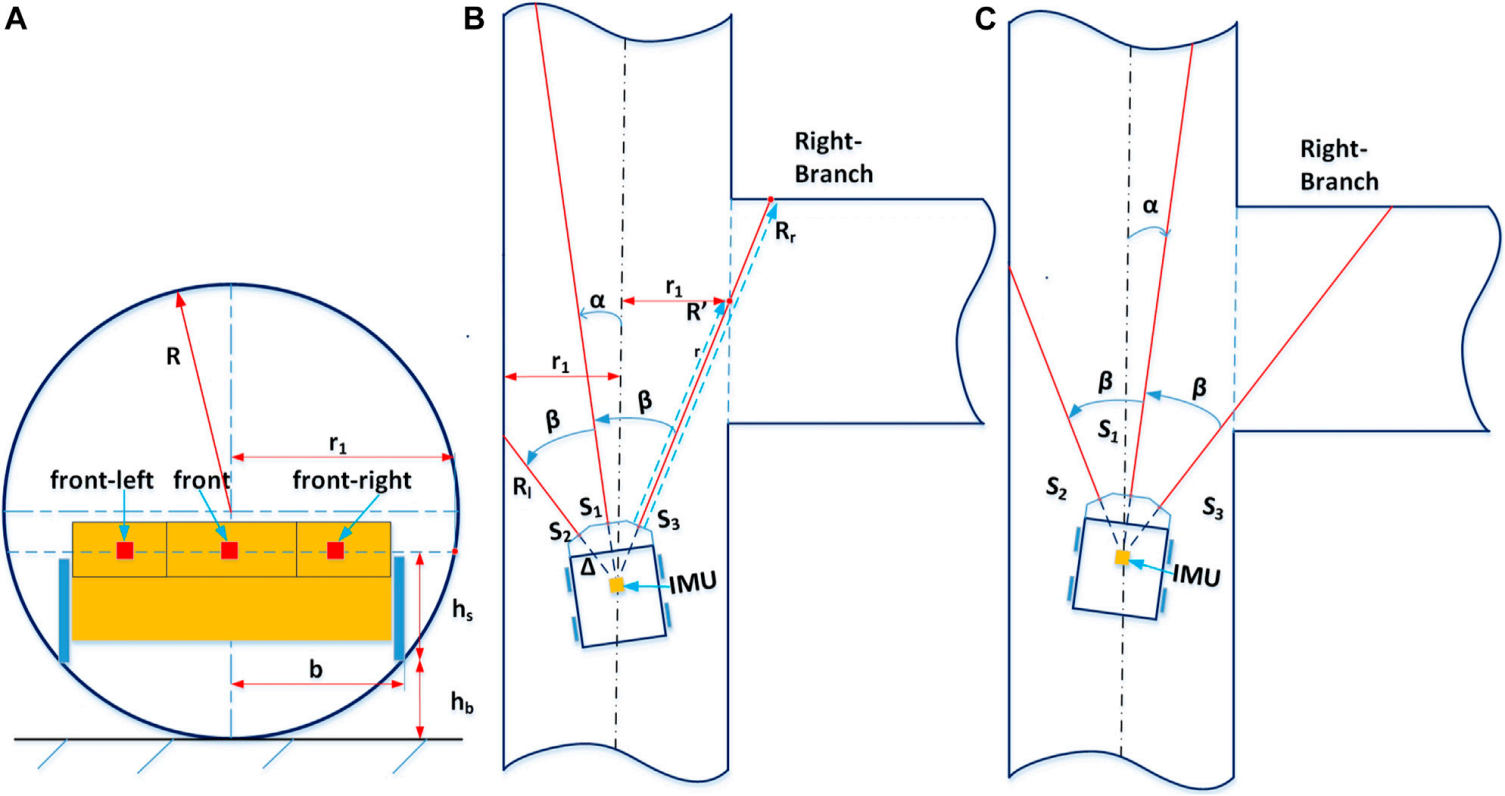
Principles of estimating robot state by range sensors. Panel **(A)** shows the relative position of the Joey robot base in the round pipe radius R. r_1_ is the half pipe width at the height that range sensor measuring ray points to. Panel **(B)** and Panel **(C)** depict the two typical orientations of the Joey approaching a right branch and the principles of determining a right branch with range sensors. In both cases, the right range value is significantly larger than the expected right range value for a straight pipe.

**FIGURE 5:**
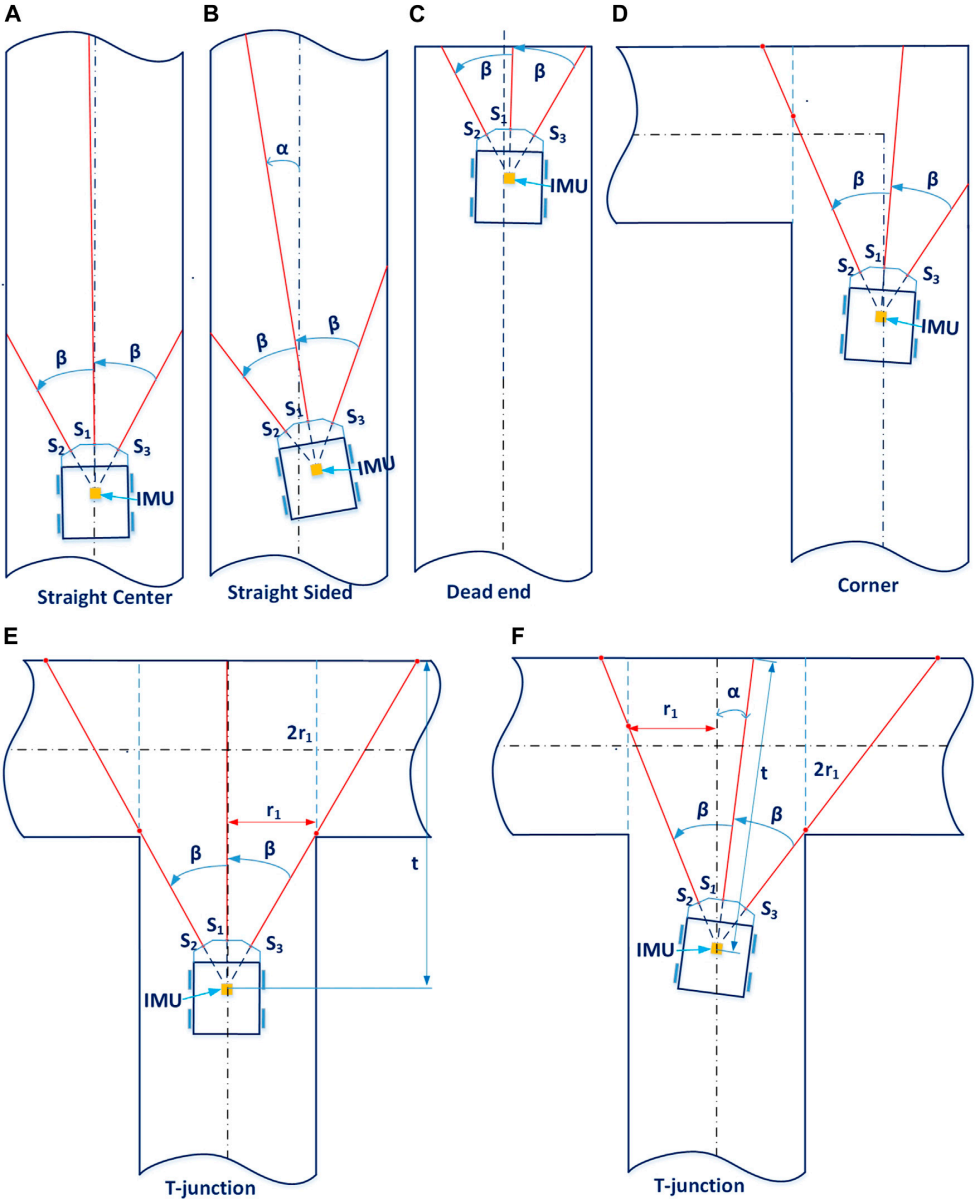
Junction classification using range sensors. Panel **(A)** and Panel **(B)** show the robot in a straight pipe, with similar left and right range values (these two states can be distinguished by checking the robot inertia data). Panel **(C)** illustrates a typical position of the robot when it detects a dead end (its front range value is under a certain limit and the sum value of its three range sensors is under a defined limit). Panel **(D)** shows the robot approaching a corner. As its left range value is significantly higher than the expected value of the straight pipe case, it can determine there is a left corner. Panel **(E)** and Panel **(F)** show the robot approaching a T-junction at a straight angle (α = 0, Panel E) or at a small angle (α > 0, Panel F). By changing this small angle α, we can determine the minimum and maximum distance $d_{A}$ (i.e., T_ASSESSING_DIS_MIN and T_ASSESSING_DIS_MAX) to assess if the robot is approaching a T-junction or corner.

Some governing rules that determine the output state of the estimation algorithm are as followed:

- If the roll and/or pitch angles are high, the robot risks flipping itself (irrecoverable).
- If the front range value is very small, the robot is about to crash into a wall/obstacle.
- If all range values are high, the robot is in an open space or manhole.
- If the front range value is just under the manhole range limit, the robot is in a straight pipe section (either at the pipe centerline or sided). It may also be approaching a right/left branch junction. The method of determining if there is a right branch is illustrated in. A similar method applies for left branch.
- The robot states of T-junction and left/right corner are only determined when the robot is sufficiently close to the junction/corner at an assessing distance. This assessing distance is calculated so that robot range sensors are able to “detect” the pipe on the left/right (i.e., the left range/right range sensor value is significantly larger than its value when it points to a pipe wall). The method of calculating this distance is illustrated in Figures 5E, F and Eq. 8.
- During a high-level maneuver, the current robot state is maintained until the robot yaw angle reaches the maneuver target (within tolerance), at which point the maneuver terminates.

The pseudo-code of the state estimation is as follows:
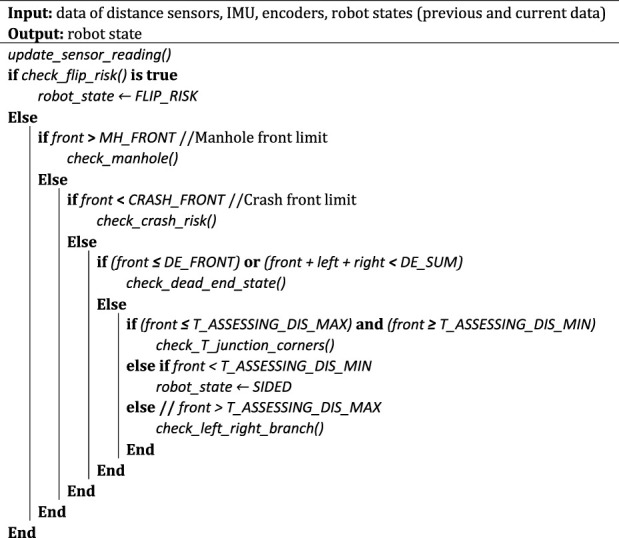


Here, MH_FRONT and CRASH_FRONT are front limits to determine if the robot is facing a manhole and or it is going to crash to a wall or object; T_ASSESSING_DIS_MIN and T_ASSESSING_DIS_MAX are the minimum and maximum front range values that the robot starts checking if it is approaching a T-junction or a right/left corner. In addition, the sub-functions are:

check_flip_risk (): reads robot pitch, roll angles and distances to nearest left, right, front objects/walls to estimate the risk of flipping over.

check_manhole (): determines the robot state when front range value is larger than the manhole front limit (i.e., MH_FRONT). Possible outputs states of this function include open space or manhole, straight center, straight sided, and left/right branch.

check_crash_risk(): calculates the robot speed and location relative to nearest wall/object when the front range value is below CRASH_FRONT or total value of three range sensors is below CRASH_SUM, and determines if it is facing a risk of crashing itself to the object/wall. Possible outputs of this function include crash left/right, dead end, and sided.

check_dead_end_state(): calculates the robot position relative to surrounding pipe walls, objects when the front range value is small but larger than front crash limit (i.e., CRASH_FRONT). Possible output states of this function include dead end, sided, T junction, and left/right corner.

check_T_junction_corners(): calculates the robot position relative to surrounding pipe walls when the robot is approaching a junction or corner at a certain front range value. Possible output states of this function include T junction, left/right corner, and left/right branch.

check_left_right_branch (): calculates the robot position relative to surrounding pipe walls when front range value is higher than the distance needed for assessing a T junction but smaller than the manhole front limit. Possible output states of this function include left/right branch and straight sided.

Limit values for the above sub-functions to determine output state are designed based on the pipe width at the height of the range sensor measuring ray points to (hereafter dubbed “virtual pipe diameter”), $2r_{1}$ , and Joey specifications, as follows:

$$h_{b}=R-\sqrt{R^{2}-b^{2}}$$ (2)

$$h={\;h}_{b}+{\;h}_{s}$$ (3)

$$r_{1}=\sqrt{2Rh-h^{2}}$$ (4)

where 2b and $h_{b}$ are the robot width and its height from the bottom of the pipe to the base of the robot, $h_{s}$ is the height of range sensors from the base, so that the sum h is the height of range sensor (i.e., its measuring ray) from the bottom of the pipe. Thus, $r_{1}$ is the pipe half width at a height the range sensor measuring ray points to. Figure 4A illustrates the robot position in a pipe from the front view. The red squares are the locations of front, front-left, front-right range sensors in front of the robot. This figure shows how the virtual pipe radius $r_{1}$ is related to pipe radius R and robot width 2b . Figures 4B,C illustrate the geometric calculation used to determine if there is a right branch ahead. As the low-level control should steer the robot to the pipe centerline, we assume that the robot is well centered within the pipe and the robot front-back axis forms only a small angle α with the pipe centerline. Then α and the expected right range $R_{r}^{\prime }$ (when the right range sensor points to a straight pipe wall without a right branch, as shown on Figure 4B) are calculated as:

$$\alpha =\arcsin\left(\frac{r_{1}}{R_{l}+\Delta }\right)-\beta$$ (5)

$$R_{r}^{\prime }=\frac{r_{1}}{\sin\left(\beta -\alpha \right)}-\Delta$$ (6)

where β is the designed angle of the left/right range sensor measuring direction to the robot front-back axis (showed in Figures 4B,C), *Δ* is the distance between the range sensor to the robot center (showed in Figure 4B). If the right range sensor output $R_{r}$ is larger than the expected right range $R_{r}^{\prime }$ plus a tolerance:

$$R_{r}>R_{r}^{\prime }+\sigma ,\;and\;\frac{R_{r}}{R_{r}^{\prime }}>\varepsilon$$ (7)

then, the controller concludes that the robot is approaching a right branch. A similar check on the opposite side applies for left branch.

Figure 5 shows how the controller determines some typical states using range sensors. Detailed values of range limits (e.g., the limits for range values of dead-end state showed in Figure 5C) that distinguish the states are available in supplementary code. Note that in the aforementioned subfunctions, besides the readings of the range sensors, the subfunction takes into account the current values and history of robot roll, pitch, yaw angles from the IMU, history of states and ranges to conclude and confirm robot states. For instance, both Figures 5A,B show the robot in a straight pipe section, and their left and right range sensors have similar values for both cases. By checking the robot pitch and roll angles, the estimator can determine which case is straight sided (Figure 5B). Figures 5E,F depict two positions of the robot approaching a T-junction: at a small angle α in Panel F and at a straight angle in Panel E. The distance for the robot to start assessing if there it is a T-junction or corner is:

$$d_{A}=\frac{\frac{r_{1}}{\tan\left(\alpha +\beta \right)}+2r_{1}}{\cos\alpha }$$ (8)

By limiting the small angle α , we can calculate the minimum and maximum of this junction-assessing distance $d_{A}$ as T_ASSESSING_DIS_MIN and T_ASSESSING_DIS_MAX, which are used in the above subfunctions.

### 3.3 Decision making: High-level control and low-level control

**High-level control**: After a robot state is confirmed, the robot chooses an appropriate action.

**Low-level control**: In parallel with high-level control rules, there are low-level governing laws that maintain robot self-balance, maintain its center to pipe centerline, avoid flip and crash risks, and change robot speed according to environmental conditions.

- During all high-level maneuvers, the robot is continuously (5 Hz) monitoring its roll, pitch angles, accelerations, and range values to assess and avoid potential risks (i.e., flipping over, crashing into wall/obstacle) and to maintain low values of robot roll, pitch, and accelerations.
- At low-level states (i.e., straight center, straight sided, left branch), the robot uses closed-loop speed control and increases speed if it is close to the pipe centerline.
- During all operations, the robot continuously monitors its range values, speed, and accelerations to avoid crashing, obstacles, and pipe walls.

**Battery control**: In addition to high-level decision making and low-level motor control, it is essential to add a power management plan to the control algorithms when the robot operates autonomously in real underground pipe networks. The main control board of Joey robot integrates a battery monitoring and charging circuit. In real operations, when the battery voltage drops below a certain level, the robot will enter a charging mode in which it either navigates to the nearest pick-up point (e.g., manhole) or to the nearest charging station. Detailed power consumption calculations were carried out during the circuit design and robot design processes. Low power components were prioritized for the design to maximize the operation time. By design, the robot can theoretically operate in autonomous mode for about 53 min. We have also tested the physical robot total power consumption in the current autonomous control mode.

## 4 Experiments

**FIGURE 6:**
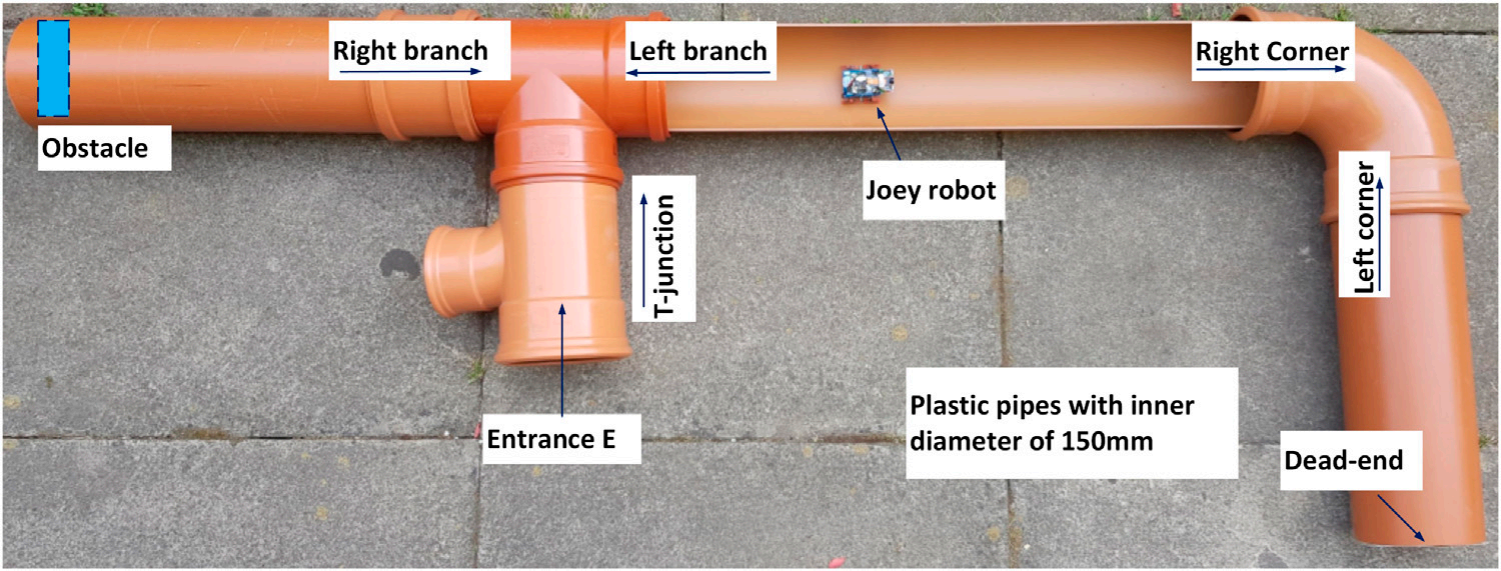
Laboratory version of small diameter (150 mm) sewer pipe network. The pipe network was constructed with a T-junction (or left branch, right branch depending on the robot moving direction as showed by the arrows), a left/right corner, a dead-end, an obstacle and three straight pipe sections. One straight section was cut half open for visual observation.

To evaluate the capability and effectiveness of the proposed control method, we tested it in physical robots in the experimental pipe network described above (Figure 2A). As specified above, the task of the micro robot was to exhaustively explore a sewer pipe network using only the low-power components described previously (i.e., without vision). Thus, the task requires the robot to move effectively in pipes without flipping over, and to navigate around the entire network. In this paper, we use two similar Joey robots as shown in Figure 1B to carry out the experiments in a pipe network as shown in Figure 6. This experimental pipe network is a laboratory version imitating the real underground drainage pipe network at ICAIR. The pipe network includes a T-junction, a left/right corner, a dead-end, an obstacle, and three straight pipe sections as depicted schematically in Figure 2A. Depending on the robot locomotion direction different robot states occur at the same position and must be correctly detected by the robot. As the Joey robot exhaustively explores this pipe network, all robot states are observed at least once except the cross junction. The total distance covered by a Joey robot during such an exhaustive exploration (passing each pipe section at least once) is 5 m.

**FIGURE 7:**
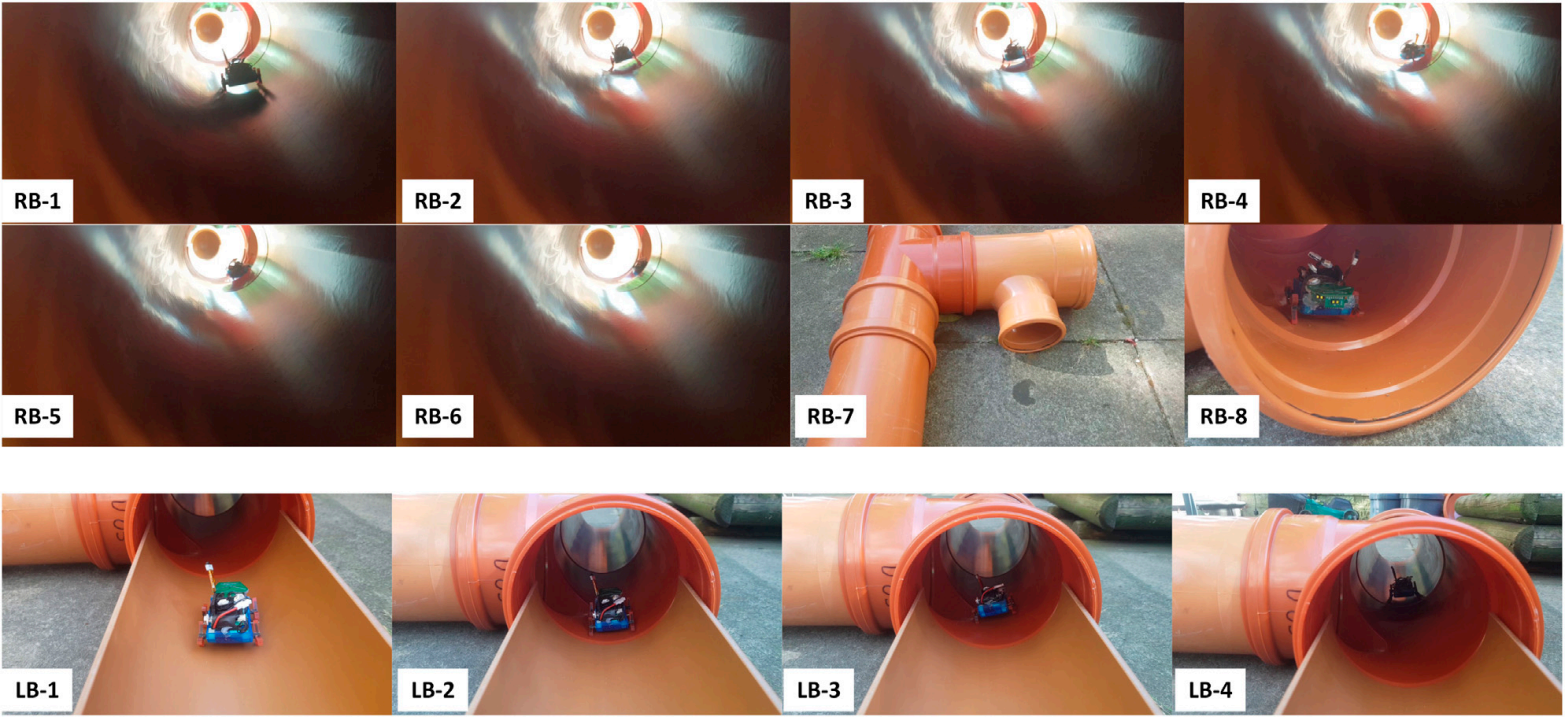
Decisions and maneuvers of the Joey robot at right and left branches during autonomous exhaustive exploration. The top two rows show in sequence (RB-1 to RB-8) images of Joey approaching and then turning into a right branch. The bottom row shows in sequence (LB-1 to LB-4) the captured images of Joey approaching but not turning into a left branch. The robot continues straight at left branch, in agreement with the high-level rule of taking the rightmost direction.

**FIGURE 8:**
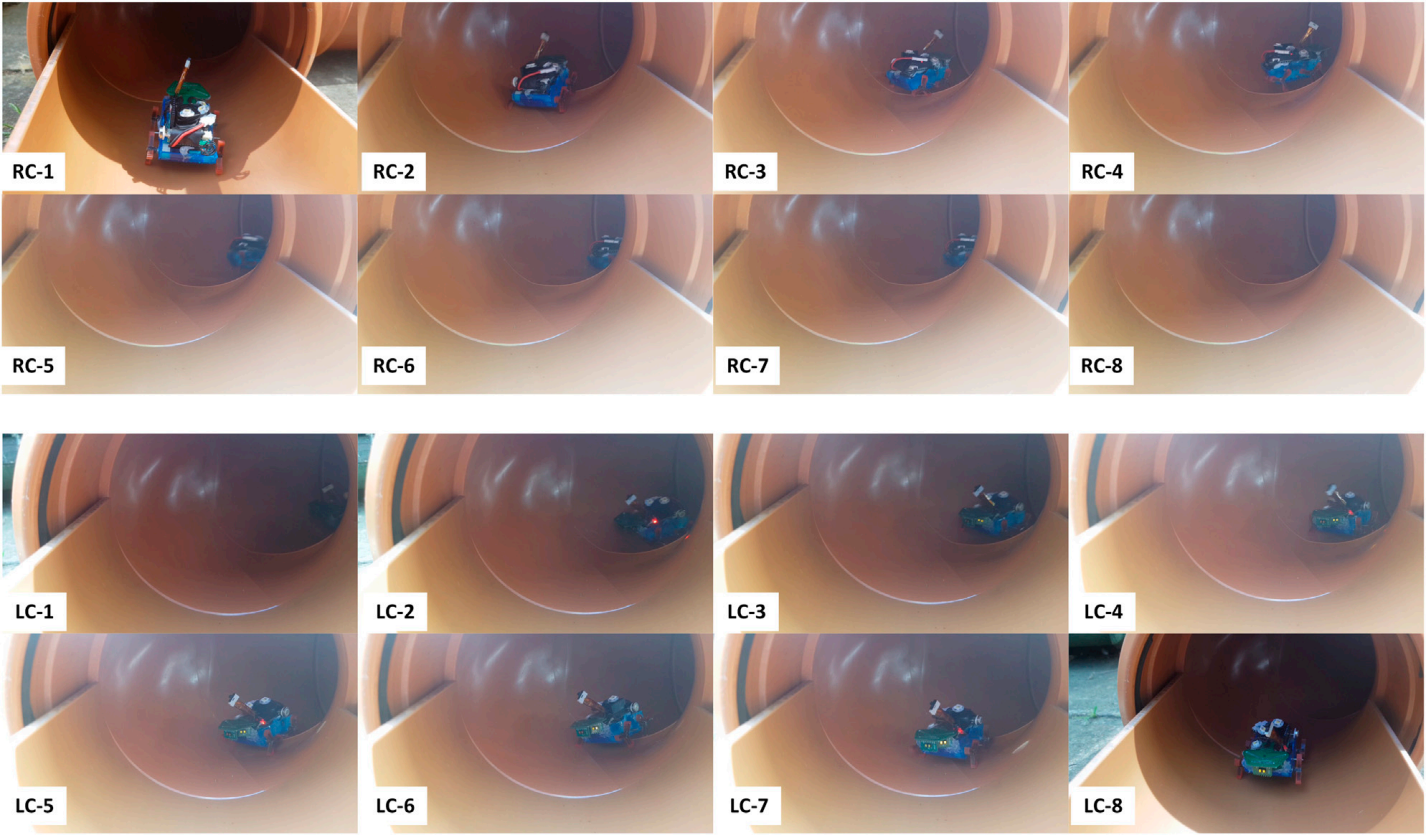
Successful detection, decision making and maneuvers of the Joey robot at right (RC-1 to RC-8, top two rows) and left (LC-1 to LC-8, bottom two rows) corners during autonomous pipe exploration.

**FIGURE 9:**
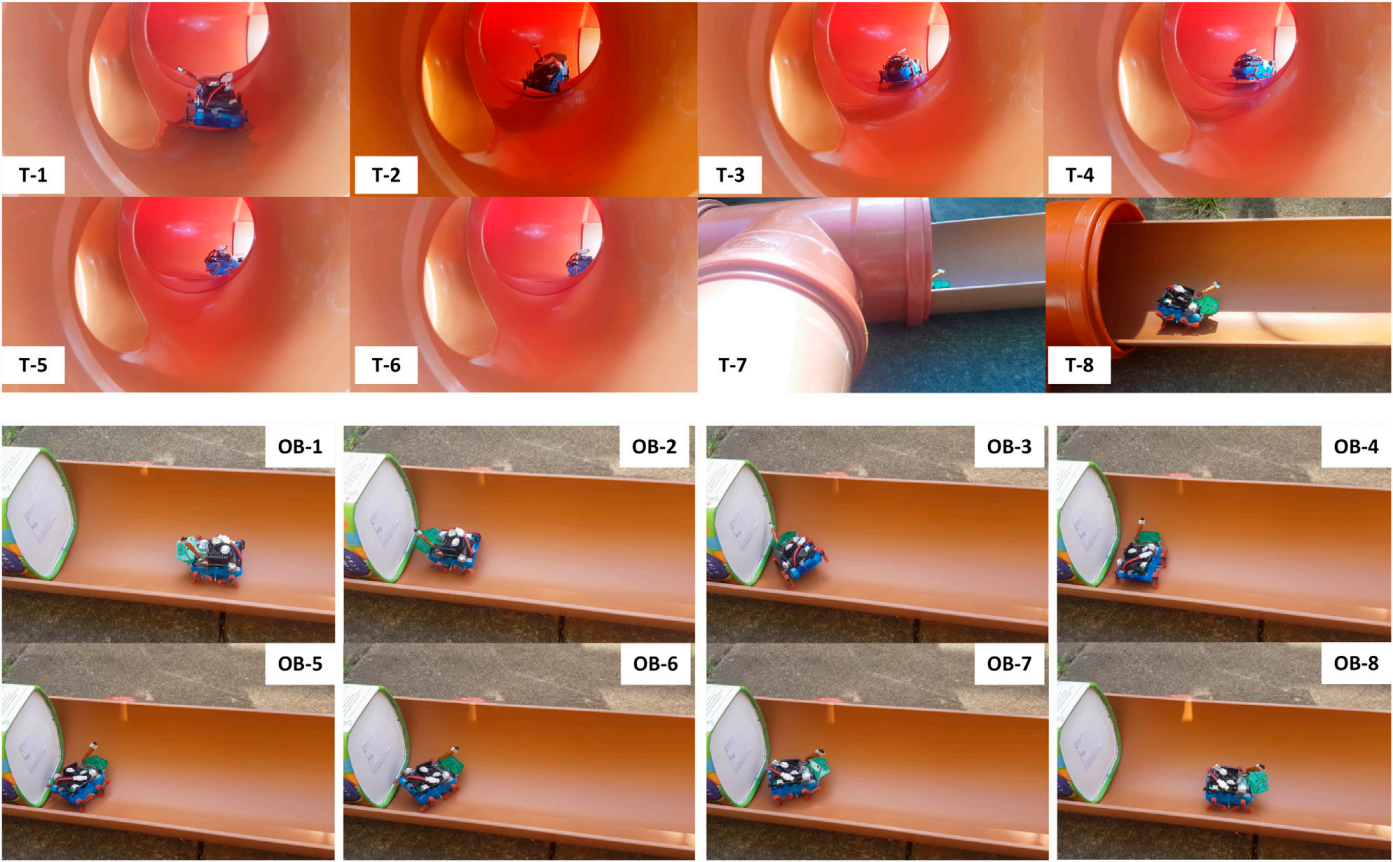
Successful detection, decision making and maneuvers of the Joey robot at T-junction (T-1 to T-8, top two rows) and large obstacle (OB-1 to OB-8, bottom two rows) in autonomous pipe exploration.

From the starting point at the entrance (Figure 2A and Figure 6), the robot walks in a straight pipe section until it reaches a T-junction. Assuming it turns right correctly at the T-junction, it walks down a long straight pipe section until it reaches a right corner. After turning right, the robot walks down a straight pipe until it reaches a dead-end. If robot turns around successfully at the dead-end, it returns along that straight pipe section until it reaches a left corner. After turning left, it follows the long straight pipe until it arrives back to the last T-junction which is now a left branch from the robot point of view. If it goes straight here, it encounters a large obstacle. If the robot detects the obstacle and turns around correctly, it approaches the right branch (previously the T-junction). The robot has exhaustively explored all sections of the pipe network if it successfully detects and turns into this right branch to return safely at the starting point. Figures 7–9 show captured images from recorded videos of the Joey robot at different points and junctions along its autonomous exploration of the experimental pipe network. Figure 7 shows the successful decisions and maneuvers at a right branch and a left branch. Figure 8 shows the successful maneuvers at the right and left corners. Finally, the maneuvers at the T-junction and obstacle are showed in Figure 9.

Additionally, extensive experiments were carried out to evaluate statistical results on high-level decision making and low-level maneuver performance by the robot when it encountered junctions, dead end, and obstacles. The Joey robot was deployed with the same sensors, components, load, and autonomous control program throughout all runs. For each junction or event, the robot was placed in front of the junction/object and a camera was used to record each autonomous run. Table 2 summarizes the results from these experiments and includes the event name, corresponding programmed action of the robot, number of experimental runs, and success rate of the robot detecting and maneuvering (requiring success both in state detection and in executing the corresponding maneuver). Recorded videos of the runs are provided as supplementary data.

**TABLE 2:** Robot single-task success rate in autonomous pipe exploration.

Event	Action	No. Of runs	Success rate (%)
Left branch	robot slightly steers away from the left branch and keeps going straight	13	85
Left corner	robot turns left 90^0^ while maintaining its center near the center of the bend, and low pitch and roll angles	11	100
T-junction	robot turns 90^0^ to the right branch while maintaining its center near the center of the junction	20	90
Right corner	robot turns right 90^0^ while maintaining its center near the center of the bend, and low pitch and roll angles	13	92
Right branch	robot moves forwards a certain distance, turns right an angle of 45^0^, then, reassesses its state, and turns to right branch	19	79
Dead-end	robot turns around by 180^0^ and adjusts its position to the center of pipe while maintaining its pitch and roll angles low to avoid flip risk	9	100
Large obstacle	robot detects a complete blockage in front and turns around by 180^0^, then explores next pipe sections	8	100
Small obstacle	robot steers away from obstacle to go in the gap between that obstacle and the other pipe wall	8	88

We recorded videos of 12 successful exhaustive explorations performed with the same Joey in the pipe network in Figure 6. The Joey robot completed exploring the whole pipe network in 3 min 50 s ± 42 s without missing any pipe sections, and without any human intervention.

**FIGURE 10:**
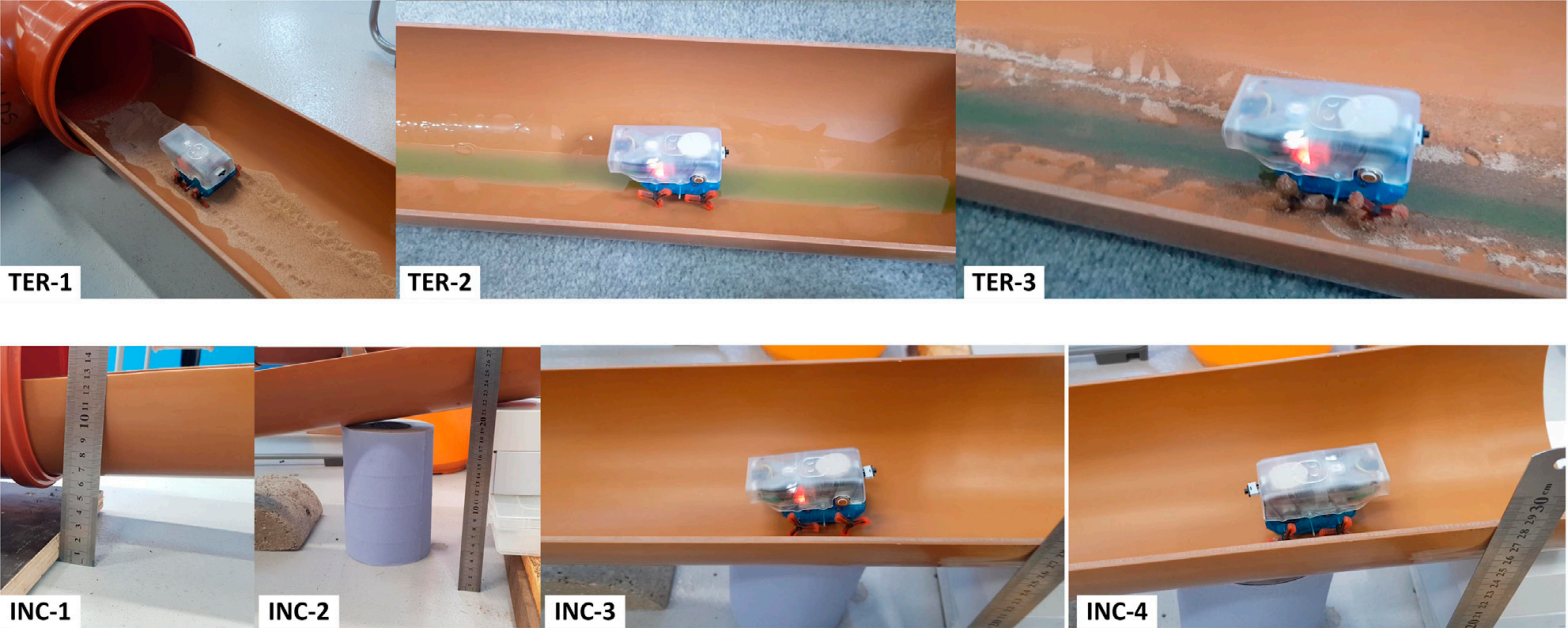
Snapshots of the Joey robot locomoting across different surfaces and on an inclined pipe. TER-1 shows the robot moving across dry sand. TER-2 and TER-3 show the robot moving along pipes with dish washing liquid, and a mixture of this liquid and sand. Lower panels: Joey robot climbing up (INC-4) and down (INC-3) an inclined pipe section shown in INC-1 and INC-2.

The above tests were performed in a horizontal and clean pipe network. Naturally, in realistic scenarios, we expect a slight inclination angle in sewer and water pipes. Worse, real pipes are rarely clean. They may contain fluids with different viscosities, and sediments such as sand. To address these challenges, we also tested the ability of the robot to autonomously locomote in the pipes under more complex and more realistic conditions, including: 1) dry sand, 2) dish-washing liquid, 3) mixture of sand and dish-washing liquid (mimicking a combination of muddy and slippery pipe conditions). In all three conditions, the miniature robot was controlled autonomously by the same pipe network exhaustive exploration program. In all repetitions of these experiments, the robot had no difficulty in locomoting and adjusting its motion to successfully traverse all three surfaces. Figure 10 (top panels) shows images of the robot captured as it moves over these surfaces.

Furthermore, we tested the ability of the robot to move up and down inside inclined pipe sections. The robot was systematically able to climb upwards and downwards without slipping on pipes with a slope of up to one in 5 (11.3^0^). In reality, sewer pipes are designed to have much shallower gradients from one in 40 to one in 110 (UK-Statutory-Instruments, 1991). This range of gradient gives adequate flow velocities to prevent any blockage, and it is not too steep to make the liquid run faster than the solids in the pipe thus leaving the solid stranded. Therefore, the Joey robot was able to climb a much steeper slope than real underground drainage pipe slope. Figure 10 (bottom panels) shows the robot climbing upwards (INC-4), climbing downwards (INC-3), and captured images of slope setup (the steepest slope tested). Recorded videos of these experiments are provided as supplementary data.

While this experimental result demonstrates the ability of the robot to effectively move through inclined pipes, if the slope in the pipe is too high, the robot may fail to estimate its state correctly (for example, recognizing left/right/T-junctions). However, we have tested the autonomous pipe exploration with the same pipe network on an incline surface with a slope of one in 10 (5.7^0^). The Joey robot was able autonomously navigate successfully without any issue.

Finally, we have tested the robot battery life while running autonomously. A fully charged robot was repeatedly deployed to run in our pipe network in the exhaustive mapping mode of operation. Each time the robot finished a full exhaustive exploration and came back to entrance, we placed it back to the entrance to start a new cycle until the motors could no longer rotate. The robot motors stopped after 31 min and traveled a total pipe length of 35 m. We use this result to plan the operation time and route for the robot to reach a safe/charging location before the battery runs out.

## 5 Discussion and conclusion

In recent years, autonomous robot operations on tough terrains have been widely developed and perfected. For instance, the Mars Rover has been functioning autonomously for years on another planet. However, these robots are big enough to carry a load of high-quality sensors, heavy battery power, and high-capacity computers. By contrast, fully autonomous control without communication, with very limited sensors and power is an unsolved challenge. This is the challenge we are solving and presenting when we deal with small, buried pipes.

This paper demonstrates an effective control method for a micro-robot autonomously exploring the inside of a water/sewer pipe network in a laboratory setting. The miniature robots present challenges for fabrication as well as for control. The wheel-legs in particular were designed to allow the robot to move through uneven terrains, but present challenges for stable and robust control, especially in round pipe geometries. As micro robotic fabrication improves, we expect a growing need for robust algorithms for autonomous control. This paper presents one such architecture that is suitable in principle for wheeled, wheel-legged, and legged robots. The robot is only equipped with low power, low-capacity microcontroller that cannot support machine learning or computer vision. This presents an additional challenge for autonomous control. Therefore, a key contribution of this paper is the ability to perform a combination of locomotion, state estimation, and decision making, autonomously in a real pipe network in laboratory conditions using only basic, non-vision-based sensors and a microcontroller.

The control method relies primarily on range sensors and an IMU and without using any visual aid methods for localization or navigation. The control is divided into two generic parts, which we dub high-level and low-level control. Low-level control is primarily aimed at achieving stability, moving to and along the center of pipes, and modulating speed. High-level control is used to detect the local pipe geometry, to determine the local robot state, and to use this information during decision making in order to navigate the pipe network. Both low-level and high-level control are sufficiently generic that we expect them to transfer over with only minor parameter changes to other robot platforms and indeed other pipe geometries and configurations.

The reliance of the navigation algorithm on range sensors and IMUs allows the robot to save battery power on camera, light, image storage and image processing. The proposed controller has overcome critical challenges caused by the design trade-offs and sacrifices required to keep the robot small (discussed in section 2.3) and controlled the robot stably, autonomously fulfilling Task 1 (Section 2.2): to control the robot movement autonomously in sewer pipes, exhaustively explore a real pipe network in laboratory settings, and avoid obstacles. In this way, the proposed control architecture separates mobility, stability and navigational tasks (all of which can be implemented with only low-cost components and low power consumption) from localization, mapping and pipe inspection tasks (that may require additional, possibly more power-hungry sensors (Worley et al., 2020; Aitken et al., 2021; Yu et al., 2021a; Yu et al., 2021b; Prisutova et al., 2022; Zhang et al., 2022)). An example of a hybrid experiment is presented by Li et al. (Li et al., 2022) in this issue. There, a simulation of the Joey, with its control architecture and the present pipe network were implemented in simulation. The control algorithm was enhanced to allow selective use of the camera in particular robot states, that was then used for localization and mapping.

The Joey robot was designed to be sufficiently small, specifically for operation in small pipes, while sacrificing components and functionality that can only be achieved with larger robots. For example, its very low weight of well under 100 g incurs a sacrifice of carrying load. As a result, small and lightweight sensors were installed to fulfil basic operating requirements. The robot is equipped with three time-of-flight distance sensors at its front. These three sensors allow the robot to perceive the local geometry and configuration of its surroundings. In this paper, the robot was programmed to combine these distance-sensor data with an IMU data and recent robot states to estimate its current state as well as to recognize if it is encountering a T-junction, right/left branch, right/left corner or an obstacle/dead-end. While the robot operates in the pipe environment effectively with a high success rate, its success rate is not 100%. The consequence of such mistakes is that the robot exploration may fail to be exhaustive (e.g., if it turns left or continues straight rather than exploring a branch to the right). Furthermore, as the number of states included in the control algorithm are limited, not every possible encounter has been accounted for in the control algorithm. For example, we can expect certain scenarios in which the robot may confuse between unusually-shaped obstacles and junctions. Many of these scenarios may be overcome with modifications or extensions to the control algorithm, while others may ultimately prove to require additional or improved sensors.

As mentioned above, this paper does not directly address the challenge of localization and mapping. In an accompanying paper (Li et al., 2022), we propose a vision-based algorithm that would allow pipe robots (and in particular the Joey robot when equipped with a camera) to perform localization and mapping in a pipe network. In that study (Li et al., 2022), Li et al. propose to only turn on the camera when at specific locations (junctions and dead-ends) to minimize power consumption. They use active vision at these select landmark locations to perform localization and topological mapping of the pipe network. We propose that this and similar hybrid solutions may provide effective and efficient solutions for the ultimate aim of these robots: to combine autonomous exploration, navigation, localization, mapping and inspection tasks over useful time scales and infrastructure sizes.

Joeys were envisioned as small robots that can operate in groups or swarms in buried pipe networks. Due to their small size, Joeys are ideal for navigating small (100–150 mm diameter) water, sewage, and drainage pipes. However, their small size, limited speed and limited stability is also prohibitive. We therefore envision Joeys being delivered and picked up by larger robots from specific locations in a pipe network. In future work, collaborative control will be developed and implemented for multiple Joey robots collaboration and for their interaction with their carriers (larger robots developed by Pipebots). Our envisioned cooperative “multi-species” ecology of robots will increase the network covering speed and be better suited to carry out complex tasks. The ultimate aim of pipe robotics is not only to navigate but also to inspect the buried infrastructure. Therefore, additional future work involving individual autonomous Joey robots will include data collection task (see Section 2.2). Immediate steps are to install the extension ESP32-CAM and program it to periodically turn on and capture images/videos at selected states or conditions, in particular at junctions or pipe sections designated for inspection and condition monitoring.

## Data Availability

The original contributions presented in the study are included in the article/supplementary material, further inquiries can be directed to the corresponding authors.
